# Trajectories of Health-Related Quality of Life and Perceived Social Support Among People Living With HIV Undergoing Antiretroviral Treatment: Does Gender Matter?

**DOI:** 10.3389/fpsyg.2019.01664

**Published:** 2019-07-23

**Authors:** Ewa Gruszczyńska, Marcin Rzeszutek

**Affiliations:** ^1^Faculty of Psychology, SWPS University of Social Sciences and Humanities, Warsaw, Poland; ^2^Faculty of Psychology, University of Warsaw, Warsaw, Poland

**Keywords:** health-related quality of life, perceived social support, people living with HIV, gender differences, latent class growth curve

## Abstract

The study examined the trajectories of health-related quality of life (HRQoL) and perceived social support (PSS) among people living with HIV (PLWH), with a special focus on gender differences. The participants included 252 PLWH (18% female) undergoing antiretroviral therapy. HRQoL (WHO Quality of Life-BREF; WHOQOL Group, 1998) and PSS (Berlin Social Support Scales; Schulz and Schwarzer, 2003) were measured three times at six-month intervals. Using a univariate approach, three trajectories of HRQoL and four trajectories of PSS were identified. Gender and relationship status were significant covariates for PSS only, with overrepresentation of single women in the increasing trajectory. The dual trajectory approach revealed a match in the decrease of HRQoL and PSS, but only for 31% of the sample. In fact, decreasing PSS co-occurred with increasing as well as stable HRQoL. There was no significant gender effect in this regard. Although a clear correspondence for decreasing trajectories exists, the findings also highlight a discrepancy between HRQoL and PSS changes that are unrelated to gender.

## Trajectories of Health-Related Quality of Life and Perceived Social Support Among People Living With HIV Undergoing Antiretroviral Treatment: Does Gender Matter?

Over the last two decades, cutting-edge progress has occurred in the treatment and prevention of HIV infection, which has not only significantly reduced mortality and morbidity among people living with HIV (PLWH; Samji et al., 2013; Cohen et al., 2016) but has also been attributed to signaling the end of the HIV/AIDS epidemic (Deeks et al., 2013; Carrico, 2019). However, the enormous progress in bio-medical care for PLWH did not directly translate to the improvement of their psychological well-being. More specifically, both in the past (e.g., Bing et al., 2001) and at present, PLWH systematically declare lower levels of well-being and higher psychological distress than the general population (Miners et al., 2014), and especially worse health-related quality of life (HRQoL) with respect to other chronic diseases (Psaros et al., 2013).

Although a large number of studies have been conducted on factors related to the HRQoL of these patients (e.g., Burgoyne and Saunders, 2001; Hansen et al., 2009; Herrmann et al., 2013; Bucciardini et al., 2014; Lifson et al., 2017; Mitchell et al., 2017; Torres et al., 2018), they failed not only to provide a consistent picture of the variables associated with the HRQoL of PLWH but also to produce a convincing answer to the aforementioned issue of low well-being among PLWH, especially at a time when their life expectancies are similar to the general population (Degroote et al., 2014). The only visible trend deals with the fact that clinical factors have been historically considered as the predominant predictor of the well-being of PLWH (Lubeck and Fries, 1997), while there is now a growing recognition of psychosocial factors as the major determinant of their quality of life (Chida and Vedhara, 2009; Cooper et al., 2017). Some authors underline that the plethora of inconclusive results in research on HRQoL among PLWH is derived from methodological shortcomings among most studies on this topic. Notably, the literature is dominated by cross-sectional frameworks using the variable-centered approach, which focuses only on the average values for an entire study sample and neglects the heterogeneity of well-being among PLWH and its relationship with sociodemographic, clinical, and psychological characteristics (Oberjé et al., 2015; Rzeszutek and Gruszczyńska, 2018). Therefore, the present study aims to overcome these methodological drawbacks by applying a longitudinal design accompanied by a person-centered perspective that allows for the identification of subgroups with different levels of HRQoL and their changes during time. Specifically, we aim to enrich the literature by focusing on the relatively understudied issue of gender-based differences in HRQoL among PLWH.

Surprisingly, although the global amount of HIV-infected women is similar to the number of HIV-infected men, both of which continue to grow worldwide (European Centre for Disease Prevention and Control/WHO Regional Office for Europe, 2017), the majority of studies on HRQoL in HIV infection were conducted on male populations only (e.g., Jia et al., 2004; Liu et al., 2006; Song et al., 2016; Emuren et al., 2017). Thus, research on HRQoL among HIV-infected women remains scarce (e.g., McDonnell et al., 2000; Gielen et al., 2001). In addition, authors examining gender differences in HRQoL in this patient group consequently observed lower HRQoL among HIV-infected women than HIV-infected men (e.g., Campsmith et al., 2003; Mrus et al., 2005; Chandra et al., 2009). There are several hypotheses on this consistent finding that have pointed to the limited access to antiretroviral treatment (ART) in some world regions (Penniman et al., 2007; Aziz and Smith, 2011), more intense HIV-related stigma, and an associated higher rate of mental disorders among HIV-infected women than HIV-infected men (Campbell et al., 2006; Machtinger et al., 2012; Geary et al., 2014). On the other hand, several studies have demonstrated that females living with HIV exhibit far greater adherence to treatment than their male counterparts and therefore report better HIV-related clinical outcomes (Collazos et al., 2007; Nicastri et al., 2007; Bor et al., 2015). In considering the aforementioned findings, it seems that no comprehensive explanation for the observed gender differences in HRQoL among PLWH exists to date. As such, the present study examines one variable that may provide important context on this topic – social support (Carvalhal, 2010).

HIV/AIDS is a chronic disease that promotes multidimensional psychological distress among PLWH, which is now primarily associated with persistent HIV-related stigma and social isolation (Rendina et al., 2018). Many studies have observed that social support – particularly perceived social support (PSS) – is one of the most important assets in coping with HIV infection and related distress (e.g., Turner-Cobb et al., 2002; Gonzalez et al., 2004; Earnshaw et al., 2015). More specifically, perceiving a high availability of support may enhance adjustment to HIV infection directly through improved adherence to treatment (e.g., Ashton et al., 2005; Alemu et al., 2012) and also indirectly through buffering the effect of HIV-related stigma on mental functioning and quality of life among these patients (Bekele et al., 2013; Breet et al., 2014).

Although the beneficial effects of perceived support on quality of life of PLWH are widely known, some authors have recently become increasingly skeptical regarding this unambiguously optimistic picture and have highlighted several methodological shortcomings of existing studies conducted using a cross-sectional framework, which is the most common (Qiao et al., 2014). Consequently, it is difficult to solve the *egg or chicken dilemma* in research on the link between perceived social support and HRQoL among PLWH. Notably, existing studies have been conducted in small samples (since the implementation of a repeated measurement design among PLWH may be challenging; Burgoyne and Renwick, 2004) or using only a baseline assessment of PSS as a predictor for HRQoL changes (Jia et al., 2005). Thus, the current literature has not examined possible heterogeneity of the dual trajectories of HRQoL and PSS. Furthermore, another topic that remains understudied in the literature deals with gender differences in both PSS and HRQoL among PLWH (Gordillo et al., 2009).

### Current Study

In considering the aforementioned research gaps, the aim of our study was three-fold. First, we aimed to examine whether heterogeneity of univariate change of HRQoL and PSS exists among PLWH, and if these trajectories are also gender-related, both with and without other possible sociodemographic and clinical covariates. Then, the probability of following a given pattern of the dual trajectories of HRQoL and PSS was explored. Specifically, we were interested in the co-occurrence of trajectories with the same direction of change, under the assumption of existing cross-sectional studies that a decrease or an increase in HRQoL corresponds to relevant changes in PSS. Finally, we examined whether any gender differences in joint probability for dual trajectories existed, supposing that combinations of changes may not be equally distributed due to gender-related patterns of social exchange as well as the social consequences of being diagnosed with HIV.

## Materials and Methods

### Participants and Procedure

The participants were 252 persons with confirmed HIV-positive results undergoing antiretroviral therapy in an outpatient clinic. The majority of them were men, which is typical based on the gender-related prevalence rate of HIV infection in Europe and the United States (Joint United Nations Programme on HIV/AIDS (UNAIDS), 2018). Detailed characteristics of the sample are provided in Table 1.

**Table 1:** Sociodemographic and clinical variables in the studied sample (*N* = 252).

Variable	*N* (%)
Gender	
Male	208 (82.5)
Female	44 (17.5)
Age in years (*M* ± SD)	39.03 ± 10.40
Marital status	
Married	147 (58.3)
Single	105 (41.7)
Education	
Basic vocational	109 (43.3)
Secondary and university degree	143 (56.7)
HIV/AIDS status	
HIV+ only	215 (85.3)
HIV/AIDS	37 (14.7)
HIV infection duration in years (*M* ± SD)	7.23 ± 6.23
Antiretroviral treatment (ART) duration in years (*M* ± SD)	5.82 ± 5.25
CD4 count	575.48 ± 2248.89

*M, mean; SD, standard deviation*.

The study design was longitudinal, with three measurements at 6-month intervals. After written informed consent was obtained from a participant, they filled in the self-descriptive questionnaire provided. For the next two measurements, they were approached during their control visit in the outpatient clinic after establishing the date *via* phone or email, based on their preference. All longitudinal data were collected by trained research assistants using a “paper-and-pencil” approach. Participation in the study was voluntary. The study was approved by the institutional ethics committee.

### Measures

*Health-related quality of life* was assessed using the WHO Quality of Life-BREF (WHOQOL-BREF), developed under a WHO initiative for cross-cultural assessment (WHOQOL Group, 1998). The tool consists of 26 items to measure four domains: physical health, psychological health, social relationships, and environment. Each item is rated on a five-point Likert scale (scores ranged from 1 to 5), and raw scores were used. Since correlations between domains in our study were stable and moderate (from 0.48 to 0.69), and followed the research indicating a possibility of assessing global HRQoL using this tool (Harsha et al., 2016), the overall indicator was obtained by summing and averaging all item scores. Higher values indicate a higher quality of life. The reliability, measured by the Cronbach’s *α* coefficient, was 0.93, 0.92, and 0.92, from the first to third wave, respectively.

*Perceived social support* was measured using the relevant subscale of eight items from the Berlin Social Support Scales developed by Schulz and Schwarzer (2003). The answers are provided on a Likert-type scale, from 1 (*not true at all*) to 4 (*entirely true*), then summed and averaged. The higher scores indicate higher PSS. The Cronbach’s *α* coefficient was 0.93, 0.92, and 0.92, for first, second, and third measurement, respectively.

### Data Analysis

We started the analysis with univariate latent class growth curve models to examine how HRQoL and PSS changed in our sample during the study period. From 1- to 5-class solutions were tested separately for HRQoL and PSS, and error variances and covariances were freely estimated across classes. The optimal solution was identified on the basis of several criteria widely identified in the literature (Nylund et al., 2007). Namely, we used Akaike as well as Bayesian information criterion (AIC and BIC, respectively), including the sample size-adjusted BIC (SABIC). The model with lower values was favored. Next, entropy as a measure of accuracy of classification was taken into consideration; in this case, the model with higher values was favored (Celeux and Soromenho, 1996). Finally, sample proportion per class was analyzed, since classes with very few individuals may be sample-specific and difficult to replicate. The practical rule is to favor a model with a fewer number of classes when at least one class has a frequency of less than 5% of the sample size (Hipp and Bauer, 2006). Time was coded as 0 for the first measurement, 0.5 for the second, and 1 for the third (Biesanz et al., 2004). Both linear and quadratic trends were explored, but we did not present them further since all quadratic terms were revealed as insignificant.

To identify covariates of trajectories, the bias-adjusted three-step analysis (Vermunt and Magidson, 2016) was implemented in order (1) to separate modeling trajectories from their relationship with other variables and (2) to correct for probabilistic classification to classes. Namely, when univariate models were established (e.g., the number, shape, and membership of trajectories were fixed), we examined whether any differences existed in predicting this membership based on sociodemographic and clinical variables. We started with gender and then added other covariates to determine if they would modify the gender effect.

In the next step, joint probabilities were computed since we used two sets of trajectories (one for HRQoL and the other for PSS). Specifically, we were interested in the probability of belonging to a given trajectory of HRQoL when simultaneously being a member of a given trajectory of PSS. Finally, by means of multinomial logistic regression, we assessed whether the probability of being a member of each combination of HRQoL and PSS trajectories was the same for women and men, both with and without additional covariates. All analyses were performed using IBM SPSS Statistics version 25 (IBM Corp, 2016) and LatentGOLD version 5.1 (Vermunt and Magidson, 2016).

## Results

### Descriptive Statistics and Missing Values

Table 2 presents basic descriptive statistics for repeated measures of HRQoL and PSS. The dropout due to longitudinal design was 41% of the sample between the first and last measurements. Missing data analysis suggested that the pattern of missingness can be treated as random (Little’s MCAR test: *χ*^2^ = 53.32, df = 54, *p* = 0.50); therefore, the option that included all available data was chosen, with missing values for indicators being handled by the maximum likelihood function (Vermunt and Magidson, 2016). Furthermore, regarding sociodemographic and clinical variables, there were also no significant differences between completers and non-completers. However, the result for gender was on the edge of significance (*χ*^2^ = 3.95, df = 1, *p* = 0.05), suggesting a tendency of higher dropout among women than men.

**Table 2:** Descriptive statistics for health-related quality of life (HRQoL) and perceived social support (PSS).

Variable	Range	Mean	SD	Skewness	Kurtosis
HRQoL					
Time 1	1.42–4.92	3.75	0.57	−0.82	0.99
Time 2	1.58–4.92	3.74	0.56	−0.59	0.61
Time 3	1.27–4.81	3.66	0.58	−0.65	1.47
PSS					
Time 1	1–4	2.31	0.68	−0.84	0.561
Time 2	1–3	2.26	0.71	−0.75	−0.37
Time 3	1–3	2.22	0.72	−0.75	−0.29

*SD, standard deviation; sample size for Time 1, Time 2, and Time 3 was 252, 201, 149, respectively*.

### Heterogeneity of Change: HRQoL and PSS Univariate Trajectories

**Figure 1:**
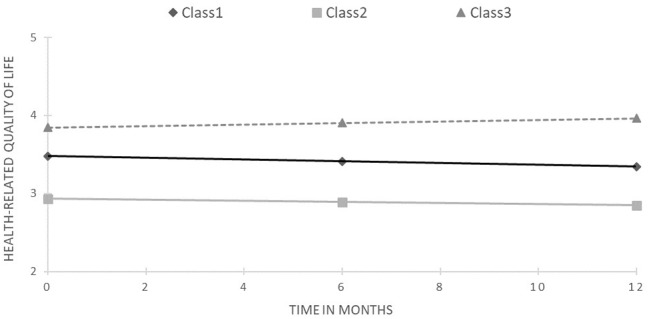
Results of latent class growth curve analysis for health-related quality of life (unconditional model).

The model fit criteria indicate that model with three trajectories was the best fitted to the HRQoL data (see Table 3). Specifically, although all the informative criteria indices scored lower with every added class, the drop in value became smaller. For models with more than three classes, the smallest class had only four members, which suggests the existence of outliners. Finally, entropy was relatively stable across all models, indicating that the perfect classification of participants was challenging. Average posterior probabilities were 0.79, 0.93, and 0.81 for classes 1, 2, and 3, respectively. This solution is plotted in Figure 1.

**Table 3:** Summary of model selection indices of latent class growth curve analysis: Unconditional univariate models for health-related quality of life (HRQoL) and perceived social support (PSS).

Model	BIC	AIC	SABIC	Number of parameters	Entropy	Smallest class	
						*N* (%)	Frequency
HRQoL							
1-Class	3467.27	3456.69	3457.76	3			
2-Class	3334.58	3309.88	3312.39	7	0.61	39.3	99
3-Class	3285.87	3247.04	3251.00	11	0.63	25.0	63
4-Class	3262.65	3209.71	3215.09	15	0.64	1.6	4
5-Class	3256.24	3189.18	3196.00	19	0.62	1.6	4
PSS							
1-Class	4039.37	4028.78	4029.86	3			
2-Class	3824.25	3799.55	3802.06	7	0.84	26.2	60
3-Class	3751.05	3712.22	3716.18	11	0.62	15.9	40
4-Class	3727.09	3674.15	3679.54	15	0.71	7.5	19
5-Class	3738.32	3671.26	3678.08	19	0.57	15.1	38

*BIC, Bayesian information criterion; AIC, Akaike’s information criterion; SABIC, sample-size adjusted BIC*.

The starting points differed significantly across trajectories (overall Wald statistics = 14101.81, *p* < 0.001; for all pairwise comparisons *p* < 0.001). The first class was the most numerous, containing 45.6% of the sample, for whom HRQoL significantly decreased during the study period (slope = −0.13, *z* = 2.55, *p* < 0.02). The second class, with the lowest and most stable HRQoL (slope = −0.08, *z* = −0.86, ns), was represented by 29.4% of the sample. Finally, the third class included 25% of PLWH and exhibited the highest and increasing HRQoL (slope = 0.12, *z* = 1.98, *p* < 0.05).

**Figure 2:**
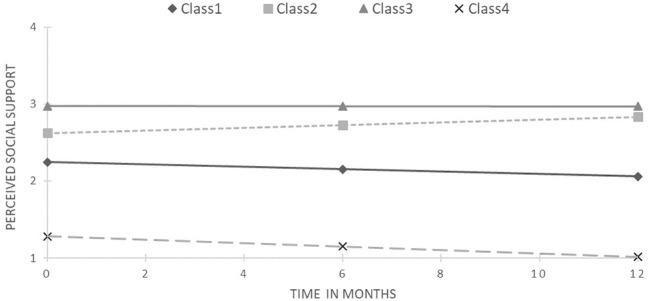
Results of latent class growth curve analysis for perceived social support (unconditional model).

For PSS, the 4-class solution was chosen as the best fitted to the data due to the lowest BIC and SABIC values, and highest accuracy of classification, while retaining a reasonable size for the smallest class. Moreover, this decision was supported by visibly worse performance on all these criteria by the 5-class model (see Table 3). Average posterior probabilities ranged from 0.78 for class 2 to 0.89 for class 1. Figure 2 presents the obtained trajectories. The majority of the sample (63.1%) was allocated to the first class and exhibited decreasing PSS trajectory (slope = −0.19, *z* = −2.21, *p* < 0.05) with a middle starting point. The second class consisted of PLWH (14.3% of the sample) with a slightly higher starting point and increasing PSS (slope = 0.21, *z* = 2.36, *p* < 0.05). The highest and stable trajectory was represented by 15.1% of the sample, identified as class 3. On the other side was the smallest class 4 (7.5% of the sample) with the lowest and stable PSS, albeit with a noticeable tendency to decrease (slope = −0.23, *z* = −1.08, ns).

### Gender as a Covariate of Univariate Trajectories

When gender was added to the models as the only predictor of class membership, it was insignificant for both HRQoL (*W* = 4.71, ns) and PSS (*W* = 3.25, ns). When all the other covariates were included in the mode (i.e., age, education, relationship status, CD4 count, duration of being diagnosed with HIV infection, duration of antiretroviral therapy and being in the AIDS stage) for HRQoL, trajectories of gender remained insignificant. Significant effects were observed for age (*W* = 7.11, *p* < 0.05) and education (*W* = 8.77, *p* < 0.02). Specifically, PLWH in the decreasing HRQoL trajectory were older than the other two trajectories (41.2 vs. 36.8 and 37.7 years of age, respectively), and PLWH in the increasing HRQoL trajectory were better educated than those with the stable HRQoL trajectory.

For PSS, both gender (*W* = 674.39, *p* < 0.001) and relationship status (*W* = 9.02, *p* < 0.05) were significant correlates of PSS trajectories. Thus, an interaction of these variables was examined, revealing a significant effect between classes 1 and 2 (*W* = 196.56, *p* < 0.001). Compared to the decreasing trajectory, there was overrepresentation of single women in the increasing PSS trajectory.

### Dual Trajectories of HRQoL and PSS: Gender Differences

Table 4 illustrates joint probability while accounting for both sets of heterogonous trajectories (i.e., for HRQoL and PSS simultaneously). It is evident that no clear correspondence exists between the trajectories of HRQoL and PSS. Although the highest probability was noted for the dual decreasing trajectory (0.31), the relatively high probability also points to the simultaneous membership of stable HRQoL and decreasing PSS. Likewise, approximately 11% of PLWH followed the increasing HRQoL and decreasing PSS trajectories concurrently. However, no members in the lowest and stable PSS reported an increase in HRQoL.

**Table 4:** Joint probability of membership for dual trajectories of health-related quality of life (HRQoL) and perceived social support (PSS).

HRQoL	PSS				
	Decreasing	Increasing	High stable	Low stable	Total
Decreasing	0.32	0.05	0.06	0.03	**0.46**
Stable	0.20	0.03	0.01	0.05	**0.29**
Increasing	0.11	0.06	0.08	0.00	**0.25**
**Total**	**0.63**	**0.14**	**0.15**	**0.08**	**1**

Finally, considering joint probability, gender differences were examined with control for all the other sociodemographic and clinical variables. We focused on the dual trajectories combining the decreasing PSS and all HRQoL trajectories, since for all the other combinations the frequency was below 10% of the sample (see Table 4). The results of multinomial logistic regression with dual decreasing trajectories as a reference category showed no significant effect of gender alone (*χ*^2^ = 3.88, df = 2, *p* = 0.14) nor of any of the remaining variables when added to the model (*χ*^2^ = 20.12, df = 16, *p* = 0.22).

## Discussion

The first aim of our study was partly achieved (i.e., we managed to observe the heterogeneity of change in HRQoL and PSS among PLWH), but no systematic gender differences were found with regard to these trajectories. Specifically, we identified three classes of HRQoL, and their members differed in terms of age and education only; therefore, clinical variables did not predict class membership. Thus, our study fits the current trend of HRQoL research, which points to the diminishing role of HIV-related clinical factors as determinants of quality of life for PLWH (Cooper et al., 2017). In a time of great progress in HIV/AIDS treatment and prevention HIV, infection has lost its fatal character and became a chronic and manageable health problem (Deeks et al., 2013). Therefore, it is unsurprising that socially valid resources such as education can be a significant predictor of HRQoL trajectories, which is in line with other studies (albeit cross-sectional and variable-centered; e.g., Degroote et al., 2014; O’Leary et al., 2014). Moreover, we observed that older age was related to decreasing HRQoL trajectories, even if they started from a relatively high level. This corresponds to existing research on elderly PLWH who compared to younger PLWH faced more problems and impediments in their daily functioning due to higher HIV-related stigma (Emlet, 2006) as well as difficulties in distinguishing physical HIV symptoms from those associated with aging (Guaraldi et al., 2011; Morgan et al., 2012).

However, one of the most important (yet null) results deals with the lack of gender differences in HRQoL change among our participants. Specifically, our finding may revise the long-lasting and relatively persistent trend in the literature, which points to lower HRQoL among female PLWH than male PLWH based on cross-sectional data only (e.g., Campsmith et al., 2003; Mrus et al., 2005; Solomon et al., 2008; Chandra et al., 2009). Some authors observed that gender differences in HRQoL within this patient group may be apparent, i.e., they disappeared after careful adjustment of the results with regard to some clinical (e.g., longer illness duration; Ruiz-Perez et al., 2009) or sociodemographic data (worse employment and education status; Rzeszutek, 2017). In other words, lower quality of life among female PLWH does not necessarily reflect their more difficult or different adjustment to HIV/AIDS in comparison to male PLWH but may rather be a result of other factors that have not been carefully controlled for in other studies (Bogart et al., 2011; Rzeszutek, 2017).

However, the results for PSS do not support this explanation. Namely, the null effect for gender became significant only when other covariates were included in the model, resulting in gender and relationship status being identified as correlates of PSS trajectory membership. For a single woman, there was a higher probability of belonging to the increasing PSS trajectory than to the decreasing one, even if the starting points of both trajectories were only slightly different. Notably, this is inconsistent with results concerning female PLWH (Li et al., 2016), but it corresponds to research demonstrating that relationship status may have different consequences for men and women, with men benefiting more from marriage (Nock, 1998). Among PLWH, men also benefit more from social support, while women are more likely to seek it (Gordillo et al., 2009; Bekele et al., 2013). Thus, being a single woman is not necessarily a disadvantageous condition in this context. Such individuals may effectively receive support from other sources that do not require HIV disclosure, and they may also be less prone to abuse from an intimate partner (Machtinger et al., 2012). However, we lack data on the relationship status of these women at the time of diagnosis; therefore, it cannot be excluded that they had been infected by a partner in a heterosexual relationship. The decision to be single could thus be a deliberate consequence of this mode of transmission – the most frequent among women in Europe and the United States (Crepaz et al., 2017; European Centre for Disease Prevention and Control/WHO Regional Office for Europe, 2017). Nonetheless, since no relevant published data exist with which to compare this gender-relationship status interaction, it could represent a sample-specific association. As such, this topic requires further research.

The obtained combinations of dual trajectories added to a complexity of change in HRQoL and PSS among PLWH. Although the probability of being a member of dual decreasing trajectories was the highest, only 31% of the sample could be assigned to this group. Thus, some factors may indeed be responsible for the simultaneous change of both HRQoL and PSS; however, they do not respond in the same manner across the entire sample, since co-occurrence of the matching change direction in both variables was rather modest. This is especially pronounced within decreasing PSS, where we identified three combinations of dual trajectories: (1) congruent decreasing PSS and HRQoL, (2) decreasing PSS and increasing HRQoL, and (3) decreasing PSS with stable HRQoL. Therefore, a few non-exclusive explanations are possible for this mixture of HRQoL and PSS change.

First, it is likely that an interrelation exists between PSS and HRQoL (Burgoyne and Renwick, 2004) causing a downward spiral over time. In this case, some general factors may broadly affect the functioning of PLWH. The natural candidates are those related to sociodemographic resources and clinical characteristics; however, this group did not differ in this respect from the other combinations of trajectories. The primary explanation for this null effect could be that we did not assess the change of these characteristics, only baselines. Nevertheless, this result is congruent with most studies, which show its modest role in the functioning of PLWH after the inception of ART (Torres et al., 2018). Second, for some PLWH, an increase in HRQoL may result in reduced perceived support since it is no longer needed to the same extent. Also, this shift in perception may additionally serve to conserve self-efficacy (Warner et al., 2011), which is of particular importance for patients with chronic diseases that have a stigmatized social reception, thereby further improving quality of life (Li et al., 2011). Finally, in the third case (i.e., for PLWH with decreasing PSS and stable HRQoL), these two processes may have different temporal dynamics, as a change in PSS likely proceeded changes in HRQoL (Jia et al., 2005). It would be rather exceptional to maintain such an incongruent dynamic-static status in light of findings that suggest cross-sectionally lower PSS is related to lower HRQoL among PLWH (Douaihy and Singh, 2001; Burgoyne and Renwick, 2004). Interestingly, being a member of each of these groups remained unrelated not only to gender but also to the other sociodemographic and clinical variables.

There are several strengths of this study, including the longitudinal and person-centered approach with a relatively large clinical sample and three measurement points. However, certain limitations must be noted. First, this is a correlational study based on self-descriptive data; thus, no causal interpretation is allowed. Additionally, the separation of the univariate trajectory classes for HRQoL was only acceptable. Moreover, since the HRQoL measurement covered social domain, significant overlap with social support is likely to occur, which may lead to an overestimation of the relationship between these variables. Even if conceptually relevant, only weak and highly similar correlation has been noted across all the domains^1^. This indicates already well-recognized difference among perceived social support, satisfaction with social support, and their correlates and outcomes (Vangelisti, 2009). Next, although the sample reflects the gender-related prevalence of HIV infection in Europe and United States, the study could be underpowered to detect gender differences. Furthermore, since there was a tendency for higher dropout among women, a recruitment bias cannot be excluded, though it was not observed in a careful examination of the general pattern of missingness. Finally, it must be underlined that the findings are restricted only to PLWH who are formally diagnosed and under medical treatment, which is a typical characteristic for most studies with clinical samples.

Despite these limitations, our study adds to the HIV/AIDS literature by investigating the heterogeneity of change in HRQoL and PSS with a special emphasis on gender differences. The present study demonstrates the complexity of dual changes and identifies groups of PLWH with mismatched HRQoL and PSS trajectories. The limited role of baseline sociodemographic and clinical characteristics of PLWH in predicting these changes was also highlighted, particularly a lack of significant differences between men and women. In fact, the findings may suggest that gender is no longer a crucial factor beyond their HRQoL and PSS change if after being diagnosed access to treatment is equal.

## Data Availability

The datasets for this study will not be made publicly available because although anonymized, it concerns sensitive issues (being infected with HIV). The informed consent did not include the consent to the publication of the data.

## Ethics Statement

All procedures performed in this study were in accordance with the ethical standards of the Research Ethics Committee of the University of Economics and Human Sciences, Warsaw, Poland, and with the 1964 Helsinki declaration and its later amendments or comparable ethical standards. Informed consent was obtained from all individual participants included in the study. The protocol was approved by the Research Ethics Committee of the University of Economics and Human Sciences, Warsaw, Poland.

## Author Contributions

EG and MR conceived the study, designed the study, supervised the data collection and database organization, conducted the interpretation of the data, drafted, and revised the manuscript. EG conducted the statistical analysis. EG and MR approved the submitted version of the manuscript.

### Conflict of Interest Statement

The authors declare that the research was conducted in the absence of any commercial or financial relationships that could be construed as a potential conflict of interest.

## Footnotes

^1^We would like to thank the anonymous reviewer for this comment. The time-averaged correlations with PSS for each domain of HRQoL were as follows: 0.25 for somatic domain, 0.40 for psychological domain, 0.40 for social domain, and 0.43 for environmental domain.
